# Sub-micrometer morphology of human atherosclerotic plaque revealed by synchrotron radiation-based μCT—A comparison with histology

**DOI:** 10.1371/journal.pone.0265598

**Published:** 2022-04-26

**Authors:** My Truong, Till Dreier, Johan Wassélius, Lena Sundius, Ana Persson, Goran Lovric, Anne Bonnin, Isabel Goncalves, Martin Bech

**Affiliations:** 1 Diagnostic Radiology, Department of Clinical Sciences Lund, Lund University, Skåne University Hospital, Lund, Sweden; 2 Department for Medical Radiation Physics, Clinical Sciences Lund, Lund University, Lund, Sweden; 3 Excillum AB, Kista, Sweden; 4 Clinical Sciences Malmö, Lund University, Malmö, Sweden; 5 Center for Biomedical Imaging, École Polytechnique Fédérale de Lausanne, Lausanne, Switzerland; 6 Swiss Light Source, Paul Scherrer Institute, Villigen, Switzerland; 7 Cardiology, Skåne University Hospital and Department of Clinical Sciences Malmö, Lund University, Lund, Sweden; Polytechnic University of Marche, ITALY

## Abstract

Histology is a long standing and well-established gold standard for pathological characterizations. In recent years however, synchrotron radiation-based micro-computed tomography (SRμCT) has become a tool for extending the imaging of two-dimensional thin sections into three-dimensional imaging of tissue blocks, enabling so-called virtual histology with arbitrary clipping planes, volumetric rendering and automatic segmentation. In this study, we present a thorough characterization of human carotid plaques after endarterectomy of patients with stroke or transient ischemic attack (TIA), investigating several different pathologic structures using both SRμCT and histology. Phase-contrast SRμCT was performed with two different magnifications (voxel sizes 6.5 μm and 0.65 μm, respectively), and histology was performed with multiple different stainings (Alpha-actin, Glycophorin A, von Kossa, Movat, CD68). The 0.65 μm high-resolution SRμCT was performed on selected areas with plaque typical relevant morphology, identified on the 6.5 μm low-resolution SRμCT. The tomography datasets were reconstructed with additional 3D volume rendering and compared to histology. In total, nine different regions with typical pathologic structures were identified and imaged with high-resolution SRμCT. The results show many characteristics typical for advanced atherosclerotic plaques, clinically relevant, namely ruptures with thrombosis, neo-vascularization, inflammatory infiltrates in shoulder regions, lipid rich necrotic cores (LRNC), thin fibrous cap, calcifications, lumen irregularities, and changes in vessel wall structures such as the internal elastic membrane. This method’s non-destructive nature renders details of micro-structures with an excellent visual likeness to histology, with the additional strength of multiplanar and 3D visualization and the possibility of multiple re-scans.

## Introduction

Stroke is the second most common cause of mortality and the most frequent cause of adult disability globally. Ischemic stroke accounts for 85% of stroke cases in developed countries and is caused by an interruption of blood flow of a cerebral blood vessel. Emboli (dislodged blood clots) from carotid atherosclerotic plaques is an important underlying cause of ischemic stroke [1, 2].

The atherosclerotic plaques are characterized by the accumulation of lipids, fibrous tissue, inflammatory cells and calcifications in the intima, the innermost layer of the vessel wall [2, 3]. The inflammatory cells that accumulated lipids often undergo necrosis. These accumulations of lipids and necrosis, often called lipid rich necrotic core (LRNC) are covered by a fibrous cap. This cap can become thinner and rupture, leading to thrombosis and the occurrence of a clinical event, such as a stroke or transient ischemic attack (TIA). Although much research focus has been directed to this field, the detailed mechanisms behind plaque formation and micro-structures architecture are not well known [2]. Specific features, such as ruptures, thrombosis, neo-vascularization, intraplaque hemorrhage, inflammatory infiltrates, shoulder regions, LRNC, disrupted endothelium, thin fibrous cap, calcifications, irregular lumen, and changes in vessel wall structures (such as the fragmentation of the internal elastic membrane), are present in advanced atherosclerotic plaques. These characteristics are essential to determine whether the plaque will rupture and cause an embolic stroke [2]. Calcifications are common in atherosclerotic plaques and visible on clinical CT if their size is in the millimeter range [4]. However, microcalcifications (<100 μm), also common in atherosclerotic plaques, are typically not visible on clinical CT due to limited image resolution [5].

Much of our knowledge of the atherosclerotic plaque microstructure is based on histology [6]. Histology has been the gold standard to visualize and analyze plaque composition. Still, plaques with calcifications and large LRNC can be challenging to section and stain due to tissue loss, laceration, fragmentation, and tissue distortion [7]. Histology is also a time- and resource-consuming method due to the multiple steps involved in processing tissue (macroscopic sectioning, fixation, dehydration, embedding, microtome sectioning, mounting on slides, rehydration, staining, imaging, and quantification) [7]. Additionally, the biochemical composition of the tissue may be affected by the histological process. In the digital era, scanning the slides to create digital images and altering hue, contrast, brightness, and balance also affects the final result.

All in all, the multiple steps between extracting the tissue at surgery up to the final assessment may affect the final interpretation of the plaque characteristics. Finally, histology offers a very high resolution in two dimensions (axial) but has limitations in displaying complex three-dimensional structures. Unless the whole plaque is sectioned serially, discerning the shape and localization of the components in a three-dimensional fashion is very time-consuming with histology.

Imaging of plaques *ex vivo* with computed tomography (CT) offers a non-destructive method to analyze plaques. Previous studies with phase-contrast micro-CT (μCT) on human carotid arteries [5, 8–11] and coronary arteries [8, 9, 12–14] have been performed on laboratory-based CT, as well as synchrotron radiation-based CT with micron resolution but to our knowledge, not with submicron voxel size. We hypothesized that a higher resolution would facilitate comparison with histology in micro-structures and provide more information.

The purpose of our study is to volumetrically image human carotid atherosclerotic plaques with submicron isotropic voxel size using SRμCT to explore the multiplanar and three-dimensionality of typical morphological features in symptomatic plaques.

## Materials and methods

### Patients

For this study, we used atherosclerotic plaques from the carotid artery bifurcation of patients that had undergone endarterectomy after a TIA or stroke.

All patients had followed clinical standards of care in establishing the plaque as the probable source of embolization (luminal stenosis > 70%). Atrial flutter and fibrillation were excluded as sources of embolization. The local Swedish Ethical Review Authority approved the study (472/2005), and informed written consent was obtained from all patients.

### Sample preparation and study workflow

**Fig 1:**
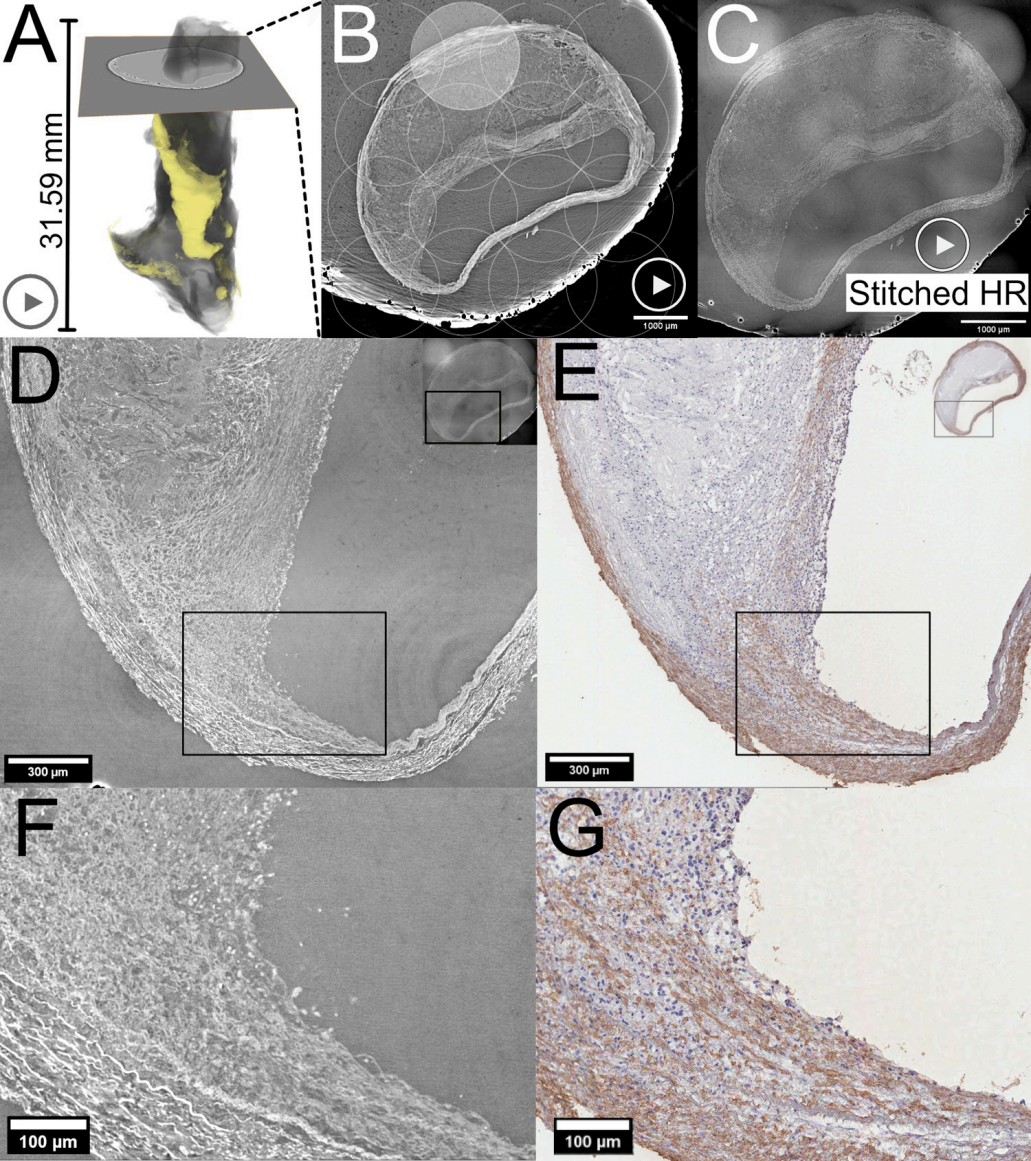
Low resolution (LR) and high resolution (HR) synchrotron radiation (SR) μCT with movie of the image sequence and 3D rendering. (A) 3D volume of LR SRμCT of a sample (calcifications in yellow) with a cross-section representing the tomographic axial plane shown in (B), where the 4x4 circles indicate the scans area of each HR SRμCT (one highlighted for demonstration). All combined they form the final stitched volume seen in (C). (D) zoomed-in area of the plaque-lumen interface on the HR SRμCT. (E) corresponding region stained for smooth muscle cells (alpha-actin). (F) magnified image of the area marked with a black square in (D). (G) magnified area within the black square in (E). The "play" symbols in (A), (B), and (C) indicate related movie material available online, showing the 3D render of the full plaque (S1 Movie in S1 File) and Movies of the LR and HR tomographic image stacks (S2 and S3 Movies in S1 File, respectively). Plaque ID 2 in S1 Table of S1 File.

For synchrotron-based imaging, the plaques had undergone typical preparation for histology without any physical sectioning. The paraffin embedded plaques were mounted free-standing such that the long axis of the sample would coincide with the tomography axis (see photographs of samples in S1 Fig in S1 File). They were scanned with multiple navigational low-resolution synchrotron radiation-based micro-computed tomographies (LR SRμCT) with 6.5 μm voxel size, for overview. The field of view (FoV) for each scan was 13.3x13.3 mm (2048x2048 pixels) in the axial plane, which covered each plaque’s full axial surface. By stacking and stitching multiple consecutive LR SRμCT reconstructed volumes, we also covered the full plaque length, which gave us an overview of each plaque. Fig 1 shows an example of the 3D volume of a plaque and the workflow of how the overview scan guided us in analyzing the HR μCT and in the comparison to histology. S1-S3 Movies in S1 File in the online supporting information, illustrate the 3D rendering, the tomographic image stack of the LR SRμCT and the HR SRμCT.

Guided by the LR SRμCT, we scanned areas that showed particular atherosclerotic pathological characteristics with multiple high-resolution synchrotron radiation-based computed tomographies (HR SRμCT) with 0.65 μm isotropic voxel size. The FoV for each HR SRμCT scan covered 1404x1664x1664 μm^3^. Multiple adjacent and partially overlapping scans at the same axial plane were reconstructed individually and stitched together to maximize the axial plane coverage (Fig 1). The factor 10 increase in resolution from LR to HR was chosen to facilitate overview, orientation, and comparison to histology since most HR SRμCT did not cover the full axial plaque diameter.

After SRμCT, the plaques were processed histologically, and the resulting stained sections scanned. Histological results were analyzed and compared to SRμCT.

### Synchrotron radiation μCT

The experiment was conducted at the TOMCAT beamline at the Swiss Light Source (SLS). We used a multilayer monochromator with the x-ray beam energy set to 21 keV. The scans were done in parallel beam geometry, and the sample was rotating over 180 degrees during the tomographic acquisition (S1 Fig in S1 File).

For the LR SRμCT, the x-ray beam was filtered by 5 mm pyrolitic graphite, 400 μm Al and 10 μm Fe. The LR x-ray detector was equipped with a 300 μm LuAG(Ce) scintillator and a 1:1 optics combined with a pco.edge 4.2 scientific complementary metal-oxide-semiconductor (sCMOS) camera (PCO AG, Kelheim, Germany) with 2048x2048 pixels giving an effective pixel size of 6.5 μm. The detector was placed 3.3 m downstream from the sample, and the exposure time was set to 30 ms. For the CT acquisition, 1501 projections were acquired as well as 30 darks and 100 flats. For each plaque, multiple LR SRμCT scans were needed to cover the entire sample. After tomographic reconstruction, the volumes were stacked and stitched along the tomographic axis to cover the full plaque length.

Following the LR acquisitions and reconstructions of each sample, selected regions of interest (ROIs) were identified. Those ROIs were then scanned with multiple HR SRμCT. The energy was still set to 21 keV for these scans, but we used a filter combination of 100 μm Al and a 10 μm Fe to increase the flux on the sample. The HR x-ray microscope was equipped with a 20 μm LuAG(Ce) scintillator, and an x10 magnification optics (Optique Peter, Lentilly, France) combined with a pco.edge 5.5 sCMOS camera with 2560x2160 pixels giving an effective pixel size of 0,65 μm. The detector was placed at a distance of 55 mm downstream from the sample, and the exposure time was set to 200 ms.

For the CT acquisition, 1501 projections were acquired as well as 30 darks and 100 flats. To fully cover the ROIs, multiple adjacent and overlapping HR SRμCT acquisitions were made. After tomographic reconstruction, the volumes were stitched together within the axial plane. The stitching procedure is described by Miettinen et al. [15]. Scan parameters are listed in S2 Table in S1 File.

### Histologic method

The samples were fixated in 4% formaldehyde for 24 hours after surgery and then placed into 70% ethanol. The plaques were embedded in paraffin for the SRμCT scans, trying to avoid air bubbles in the paraffin. After SRμCT, the paraffin-embedded plaques were prepared to remove the paraffin. They were macroscopically cut with a PAX Jewelers saw model TFS-JS (Thomas Flinn & Co, Sheffield, UK). Tissue loss of the saw was 0.254 mm at the cutting plane. The plaques were divided into 2–5 mm-thick fragments, re-embedded in paraffin, and sectioned to 6 μm in the microtome.

The LR SRμCT guided us to select section planes, and we aimed at acquiring sections visually congruent with HR SRμCT axial planes. Sections were viewed in a light microscope alongside microtoming and continuously compared to the SRμCT images. When the histologic section’s overall morphology showed visual congruency with the corresponding level plane on LR SRμCT and HR SRμCT, 6μm thick sections were saved for different stainings.

### Immunohistochemistry and histology

The primary monoclonal antibody mouse anti-human CD68, clone KP1 (DakoCytomation, Glostrup, Denmark), was used for macrophage staining. To detect vascular smooth muscle cells, a primary antibody monoclonal mouse anti-human smooth muscle cells actin clone 1A4 (Dako Cytomation, Glostrup, Denmark) was used. We used the primary antibody monoclonal mouse anti-human glycophorin A (CD235a) clone JC159 (DakoCytomation, Glostrup, Denmark) to visualize erythrocytes. For the assessment of calcifications, the Von Kossa stain was used. For collagen assessment, reticular fibers, elastic fibers, ground substance, fibrin, mucin, nuclei, and muscle, Movat Pentachrome stain was used. Technical details of the immunohistochemical and histological process are described in the supporting information.

All stained sections were scanned in 20x magnification with Aperio ImageScope 12.3.2.8013 (Leica Biosystems Inc. Buffalo Grove, IL, USA)

### Image processing and analysis

The digital histological images were viewed in Aperio ImageScope 12.4.3.5008 (Leica Biosystems Inc. Buffalo Grove, IL, USA), and relevant areas were exported as TIFF to be visually matched with SRμCT.

The SRμCT scans were reconstructed using Paganin phase-retrieval [16] with *δ* = 3.7×10^−8^ and *β* = 1.7×10^−10^ while considering the different propagation distances of the low and high-resolution setup and finally stitched together.

The low-resolution volumes were stitched along the tomographic axis after reconstruction and phase-retrieval by identifying the overlap in adjacent reconstructed volumes and combining them into one large continuous volume.

The reconstructed HR SRμCTs were stitched in the axial plane using a program developed by Miettinen et al. [15].

All stitched LR SRμCT scans for each plaque were analyzed in Fiji analysis software (National Institutes of Health NIH, Maryland, USA) [17] as 16 bit TIFF sequences and viewed as image stacks.

All stitched HR SRμCT scans were saved in TIFF image sequences. Areas with typical plaque pathology were selected, compared to histology, and sub-volumes were selected for movie compilation and volume rendering.

Segmentation of the LR and HR SRμCT volumes was performed using the Amira software (Thermo Scientific, Waltham, USA). Volumes used for visualization were downscaled for a smoother workflow in Amira. Segmentation was performed on a stitched low-resolution SRμCT by first separating paraffin and tissue from air using thresholding. Paraffin and tissue were then segmented manually in multiple steps using the ’magic wand’, ’brush’, and ’interpolation’ tools. Calcifications were segmented using a simple threshold. For the high-resolution SRμCT volumes, volumes of interest were pre-selected and cropped before import into Amira.

## Results

### Patients

Five plaques were collected from three patients with a transient ischemic attack (TIA) and two patients with stroke. One of the five patients was a female. The mean age was 72 years (range 52–83). None had diabetes. Hypertension was found in three of the patients, and three of the patients were smokers. Informed consent was obtained from all patients.

### SRμCT in low and high resolution

All five plaques were scanned in LR SRμCT, with the number of stitched scans ranging from 4 to 11. S1 Table in S1 File shows the number of stitched LR SRμCT scans for each plaque. Fig 1 shows an example of the LR SRμCT of a plaque (A and B). The movies linked to this Fig; S1-S3 Movies in S1 File, show 3D rendering and the tomographic image sequence in the axial plane of both LR and HR SRμCT. The zoomed-in HR SRμCT images and histology in Fig 1D–1G demonstrate an example of the interface between plaque and lumen with great visual detail.

Nine different plaque regions were imaged with 0.65 μm voxel size stitched HR SRμCT. The data of the image volumes are listed in S1 Table in S1 File. Each stitched HR volume contained one or multiple plaque typical morphological changes. Plaque 1 was scanned over three distinct regions, and each region was scanned with 2x2 adjacent and partially overlapping HR SRμCT scans, which were subsequently reconstructed and then stitched. Plaque 2 was imaged on two different regions—one with a region covered by 4x4 stitched scans, the largest in the study (Fig 1). The other region in plaque 2 had coverage of 2x2 scans. Plaque 3 was scanned over an area covered by one single scan. Plaque 4 had a region covered by 2x2 scans. Plaque 5 was imaged over two separate regions, each covered by 2x2 scans.

### Assessment with SRμCT and histology

Fig 1, including S2 and S3 Movies in S1 File, displays the axial plane of LR SRμCT and HR SRμCT of a plaque. The LR SRμCT with 6.5μm voxel size allowed us to scan the entire length of the plaque and navigate in multiplanar reconstruction. This facilitated both the sectioning process and the preliminary comparison with histology, while the final comparison was made with HR SRμCT. For the plaque in Fig 1, 4x4 HR SRμCT scans were stitched, and the final result covered the full plaque area on the axial plane. The stitching of multiple scans rendered a large area that covered multiple typical plaque features recognizable by histology but with the advantage of no tissue loss, change of plaque shape, or plaque configuration.

The following features, well known to be associated with advanced atherosclerotic plaque, were observed in SRμCT and confirmed in histology: ruptures, thrombus, neo-vascularization, inflammatory infiltrates, shoulder regions, LRNC, plaque-lumen interface including the irregular endothelial surface, thin fibrous cap with irregular thickness, calcifications and fragmentation of the internal elastic membrane.

### Rupture with thrombus

**Fig 2:**
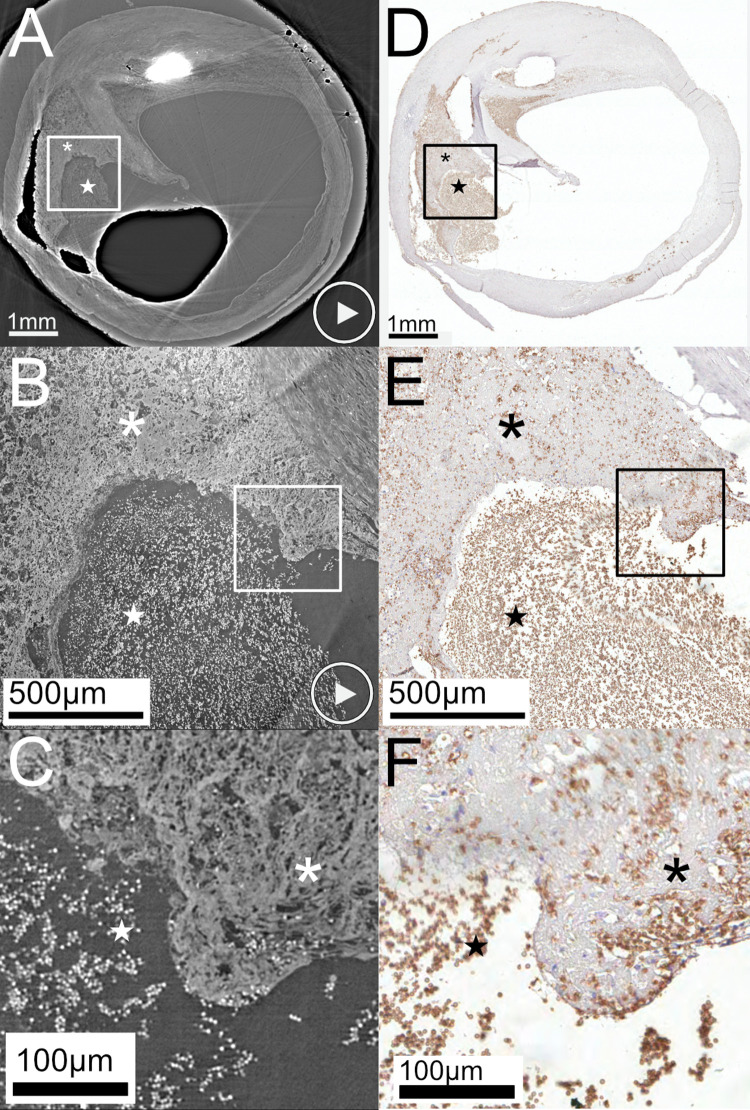
Rupture with plaque tissue exposed to the bloodstream with thrombus formation. **(A)** LR SRμCT of a ruptured shoulder region with plaque tissue (asterisk) exposed to the bloodstream with the formation of a thrombus (star). **(B)** the zoomed-in area of the interface between plaque tissue and thrombus, obtained with HR SRμCT. The white square in **(A)** corresponds to the area in **(B)**. **(C)** shows the area of the white square in B with the zoomed-in region of the plaque tissue (asterisk) and thrombus (star) interface. Individual erythrocytes are also seen scattered within the exposed plaque tissue, which is also seen in histology (D-F). The corresponding section in histology, stained for erythrocytes (Glycophorin A), is seen in **(D)**, with the black squares marking zoom-in regions in **(E)** and further in **(F)**. The black asterisk indicates plaque tissue and the black star marks the thrombus. The "play" symbols in **(A)** and **(B)** indicate related movie material, showing the LR (S4 Movie in S1 File) and HR (S5 Movie in S1 File) tomographic image stacks. Plaque ID 1 in S1 Table in S1 File.

In Fig 2, a rupture site stained for erythrocytes (Glycophorin A) is shown and compared to LR SRμCT and HR SRμCT. The rupture has caused a flap of the fibrous cap to protrude into the lumen, exposing plaque tissue to the circulation with thrombus formation. The full plaque volume, including the rupture, is imaged with LR SRμCT (A) and also shown with a movie of the tomographic image sequence (S4 Movie in S1 File). Fig 2 includes zoomed-in images (B) of the interface between exposed plaque tissue and the thrombus with a resolution that allows us to see individual erythrocytes on the HR SRμCT (also seen on S5 Movie in S1 File). The level of detail of the HR SRμCT also allows visualization of individual erythrocytes scattered within the exposed plaque content due to the structural difference between the individual cells and the surrounding tissue. This is also seen on histology from the corresponding level.

### Organized thrombus and neovessels

**Fig 3:**
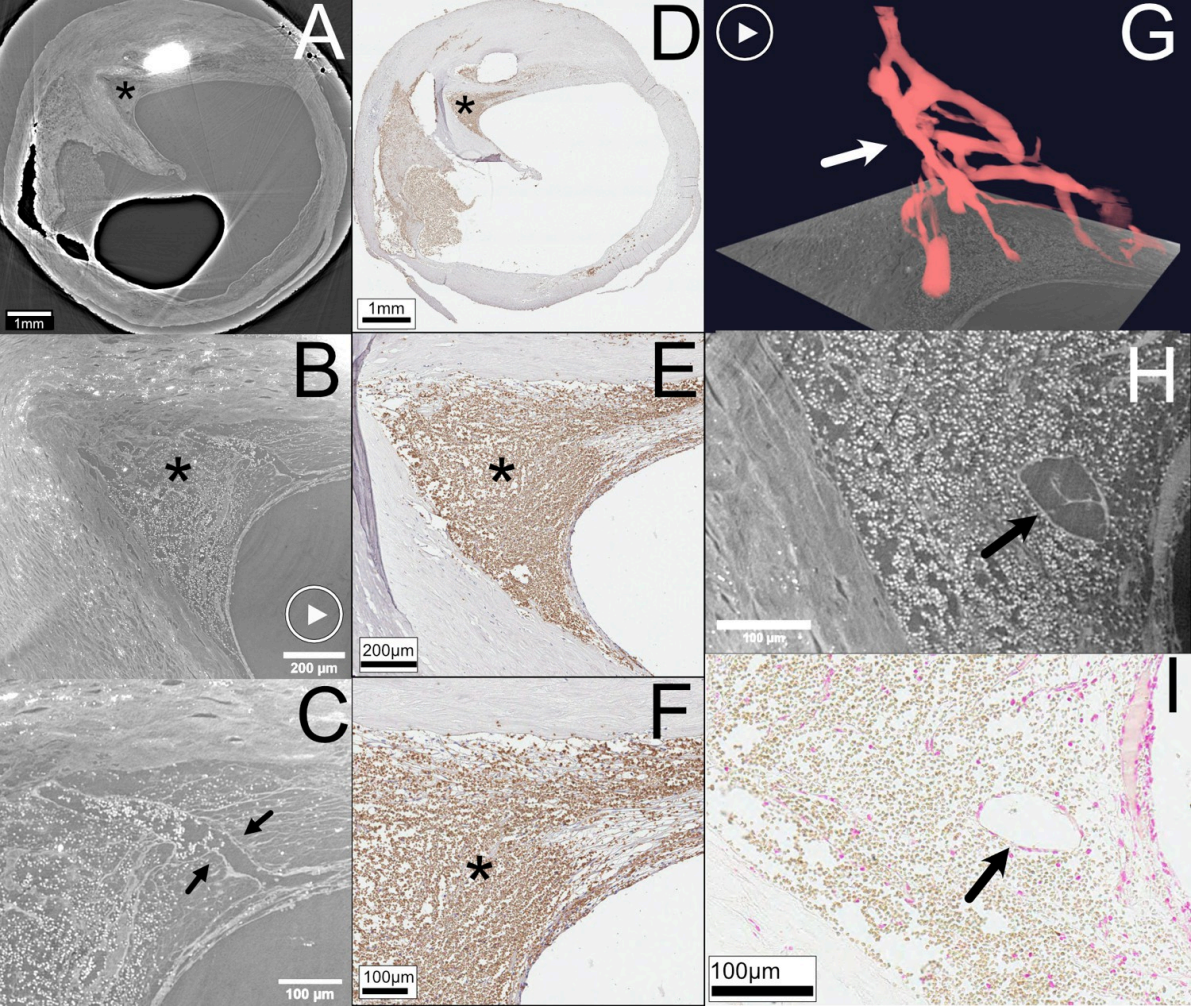
Organized thrombus with recanalization (with 2D and 3D rendering). **(A**) shows LR SRμCT with the full axial plane of a ruptured plaque, with the black asterisk indicating the organized thrombus. In **(B)** and **(C)**, the thrombus is seen zoomed-in with HR SRμCT with the neo-vessels indicated with black arrows in **(C)**. The corresponding histologic section stained for erythrocytes (Glycophorin A) is seen in **(D)**. In **(E)** and **(F)**, the thrombus area is magnified and marked with a black asterisk. **(G)** depicts a 3D reconstruction of the neo-vessels highlighted with red (arrow). In **(H)** a detailed area in the organized thrombus seen with HR SRμCT is shown with the black arrow indicating one of the neo-vessels with a matching location on a histological section (Von Kossa staining) seen in **(I)**, where the black arrow indicates the neo-vessel. The "play" symbols in **(B)** and **(G)**: movie material available online, showing the HR tomographic image stacks (S6 Movie in S1 File) and the 3D render of the segmented and highlighted neo-vessels (S7 Movie in S1 File), respectively. Plaque ID 1 in S1 Table in S1 File.

In addition to the thrombus at the rupture site seen in Fig 2, the same plaque also presented a separate area of organized thrombus with recanalization shown in Fig 3. The area of the organized thrombus contains strains of tissue covering the interface between the thrombus and lumen and within the thrombus itself. The separate Movat staining (Movat not shown in the Fig) confirms the presence of fibrous tissue. HR SRμCT allows good visualization of the complex network of neo-vessels traversing the thrombus seen in a 2D image stack (S6 Movie in S1 File). The high-resolution images of the vessels were also rendered in 3D, where the neo-vessels were segmented and highlighted with red (S7 Movie in S1 File). In this example, the complexity of the recanalization’s architectural layout is more difficult to discern in histology but easily visualized in the 2D and 3D reconstructions. The HR SRμCT image sequence conveys the complex network of vessel paths and vessel diameter’s irregularity. The histologic section shows the erythrocytes within the neo-vessels. The intraluminal localization of erythrocytes is also readily seen on HR SRμCT (C in Fig 3). Some cells have no contact with the vessel wall, and some cells are adjacently lining the endothelial surface.

### Shoulder region and inflammatory cell infiltrates

**Fig 4:**
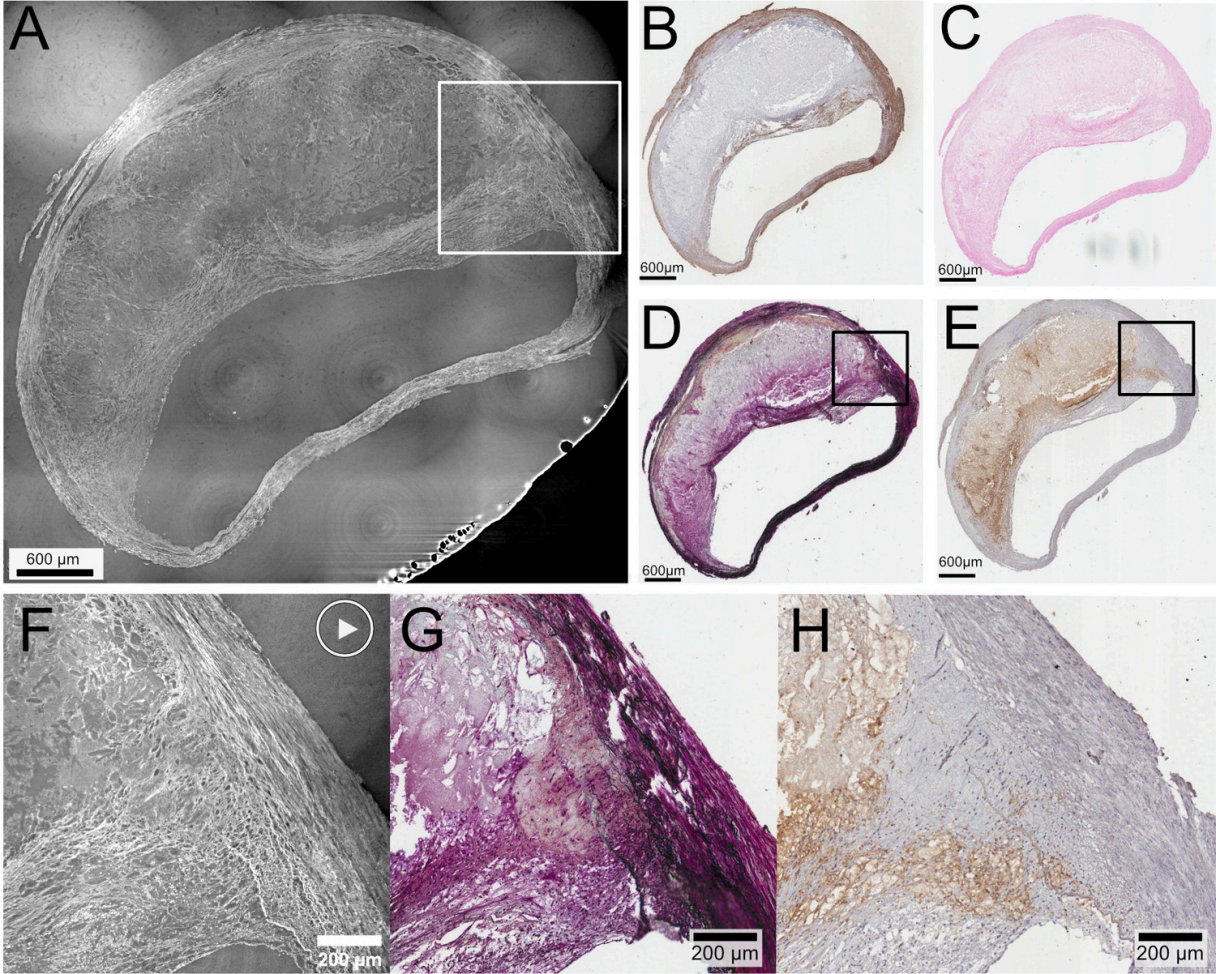
Stitched HR SRμCT showing full axial plane of plaque with the shoulder region zoomed in (white square in A, black square in D and E). Full axial plane of plaque with stitched HR SR μCT **(A)** and corresponding section plane in histology with four different stains on consecutive sections. **(B)** Alpha actin staining for smooth muscle cells. **(C)** von Kossa staining for calcifications (no calcifications present). **(D)** Movat staining for collagen, elastic fibers, nuclei, fibrin, mucins. **(E)** CD68 for macrophages. The box in **(A)** indicates the shoulder region, zoomed in on HR SRμCT in **(F)**. **(G)** and **(H)** are the corresponding area shown with the black boxes in **(D)** and **(E)** for the Movat and CD68 staining. The "play" symbol in **(F)**: online movie material (S8 Movie in S1 File) showing the HR tomographic image stacks of the shoulder region.

Fig 4 shows the full axial plane of a plaque in HR SRμCT, with the corresponding level in the histology of four different stains (alpha-actin (Fig 4B), von Kossa (Fig 4C), Movat (Fig 4D), and CD68 (Fig 4E), and zoomed-in images of the shoulder region on HR SRμCT, as well as on histology stainings for macrophages/inflammatory cells (CD68), and fibrosis (Movat).

The shoulder region is readily seen on the HR SRμCT, and the transition between the inner parts of tunica media, the fibrous cap, and the LRNC can be discerned. A very small part of tissue towards the inner part of tunica media can be seen on HR SR μCT, and the complex 3D structure of the layers is conveyed with clarity in the 2D image stacks (S8 Movie in S1 File). The interface between the loose tissue in LRNC and the dense tissue towards the tunica media and the fibrous cap can also be discerned on the HR SRμCT. Individual cells scattered within the plaque shoulder seen on HR SRμCT corresponds with inflammatory cell infiltrates seen on the CD68 stain. The S8 Movie in S1 File also conveys the complexity of the three-dimensional architecture with the gradually changing interface between different parts of the shoulder region and in the structures bordering the cap and shoulder. Plaque ID 2 in S1 Table in S1 File.

### Lipid rich necrotic core (LRNC)

**Fig 5:**
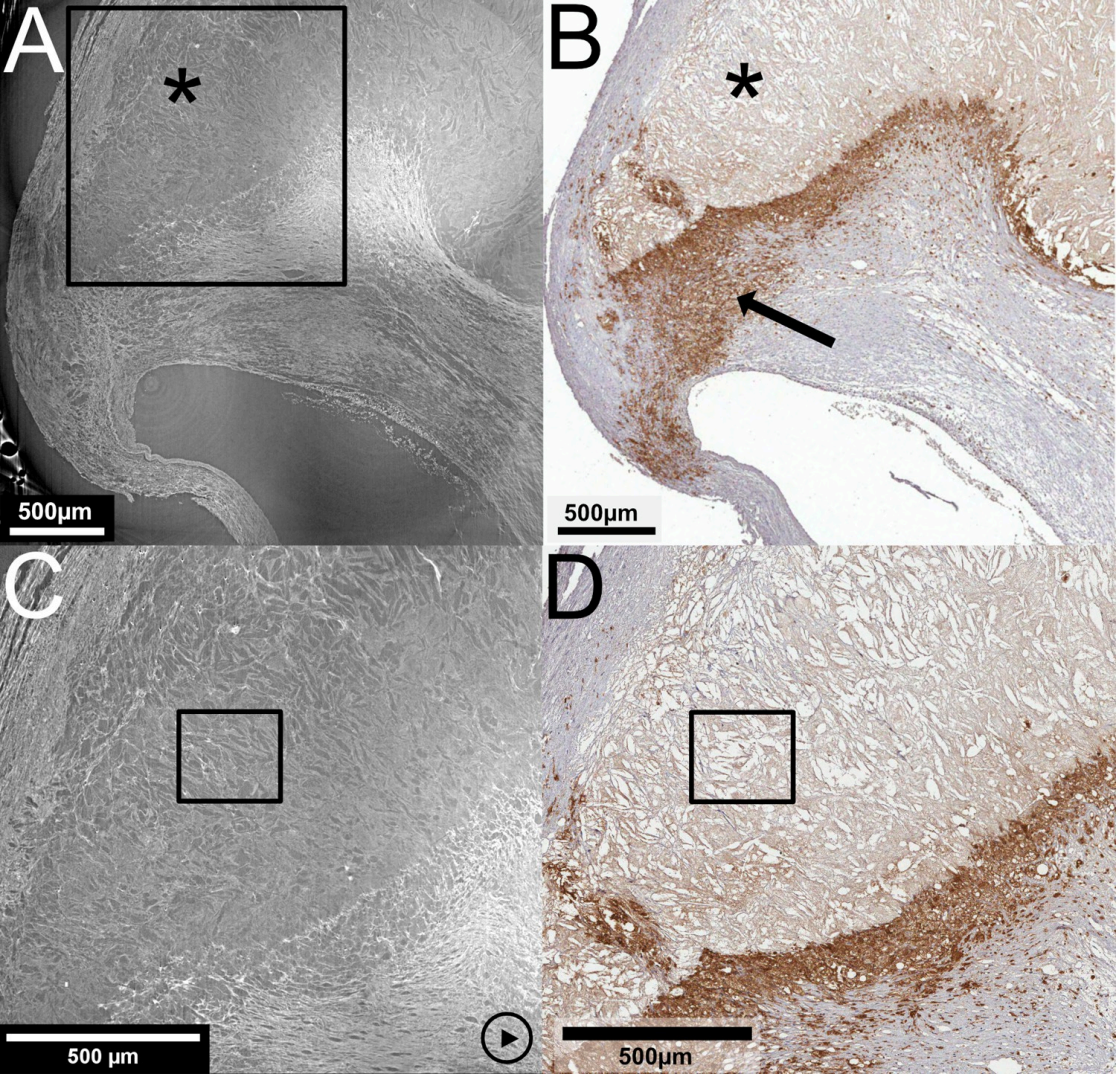
Plaque with lipid rich necrotic core (LRNC) and with cholesterol crystals. LRNC depicted with stitched HR SRμCT **(A)** and histology stained for macrophage with CD68 **(B)**. A part of the LRNC is indicated with asterisks (*). The black arrow in **(B)** points to a region dense with macrophages. **(C)** and **(D)** are zoomed in and marked with squares representing regions rich in cholesterol crystals. The "play" symbol in **(C)**: movie material (S9 Movie in S1 File) showing the HR tomographic image stacks of the LRNC with focus on the cholesterol crystals. Plaque ID 2 in S1 Table in S1 File.

Multiple areas with LRNC with features typical of cholesterol crystals were identified. Fig 5 shows an area with LRNC and adjacent shoulder region. The LRNC and the shoulder region are infiltrated with macrophages (CD68 stain). Although the histologic section is not specifically stained for lipids, the core structural content has the typical appearance of a LRNC with cholesterol crystals readily seen on HR SRμCT and the CD68 stain.

### Calcifications

All five plaques contained macrocalcifications (>100μm), which lacerated the tissue to a various extent during sectioning. In contrast micro-calcifications (< 100μm), also present in all plaques, caused fewer lacerations.

**Fig 6:**
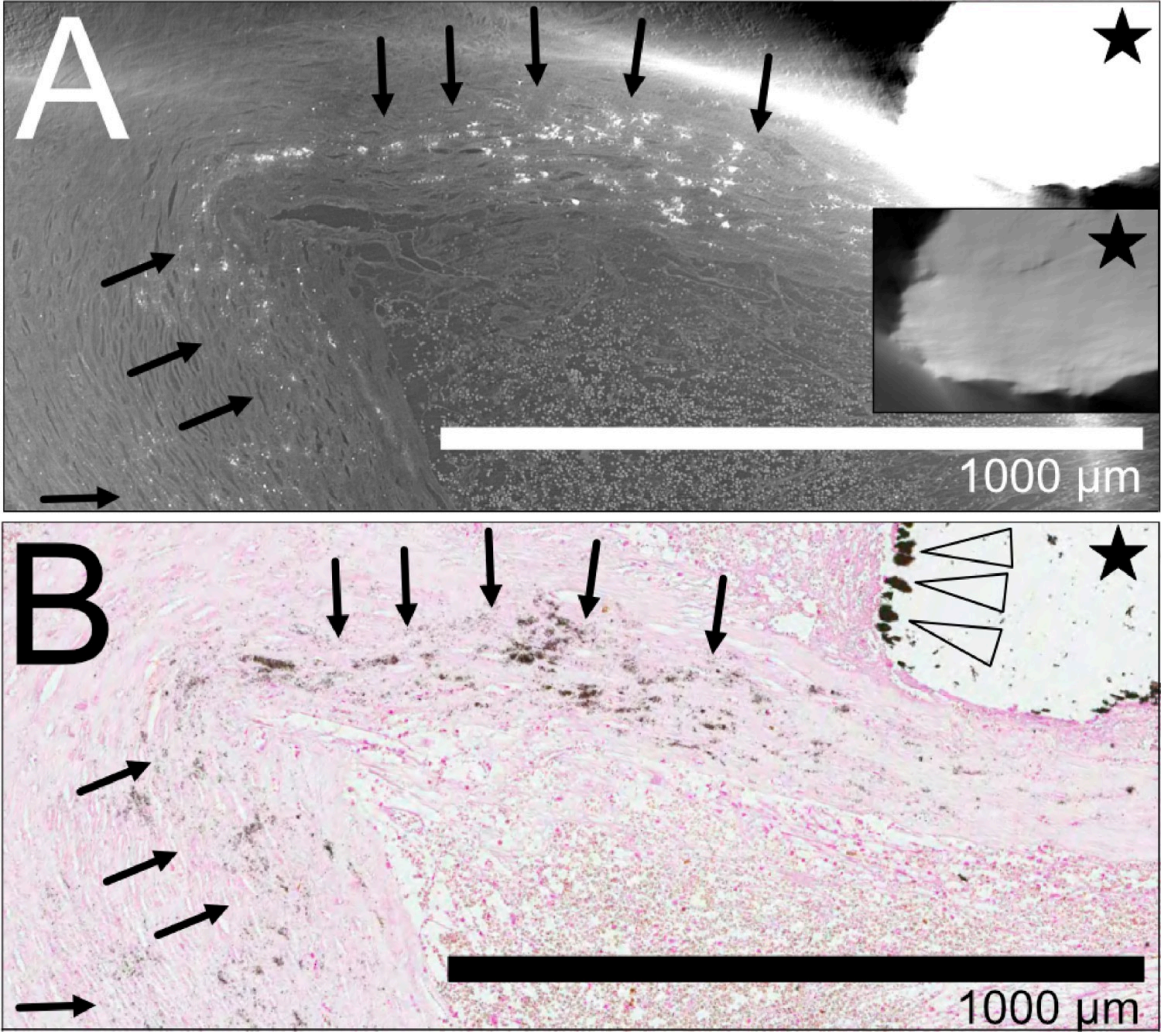
Calcifications. The black star in **(A)** indicates part of a macrocalcification in the HR SRμCT where regions of high density appears as saturated. With a wider dynamic range of the grey levels, more details are visible within the calcification (inset in **(A)**). **(B)** histological section of the corresponding area, stained for calcifications (von Kossa), where only the macrocalcification’s fragmented rim remains after the histologic preparation (arrowheads). In **(A)** and **(B)**, the black arrows indicate microcalcifications. Plaque ID 1 in S1 Table in S1 File.

In Fig 6, both microcalcifications in the fibrous cap and a macro-calcification are demonstrated. The image artifacts caused by high sample density and the over-saturation of the large calcifications jeopardize the evaluation of details within the macro-calcification, but this was partially corrected by increasing the reconstruction’s dynamic range. With this approach, details within the macro-calcifications, information usually lost in histology, can to some extent be retained. We also found examples of calcifications on HR SRμCT, that have become dislodged from the tissue on the corresponding histologic section. This exemplifies one of the strengths of SRμCT in showing the distribution of calcifications in comparison to histology.

### Fibrous cap and irregular lumen

**Fig 7:**
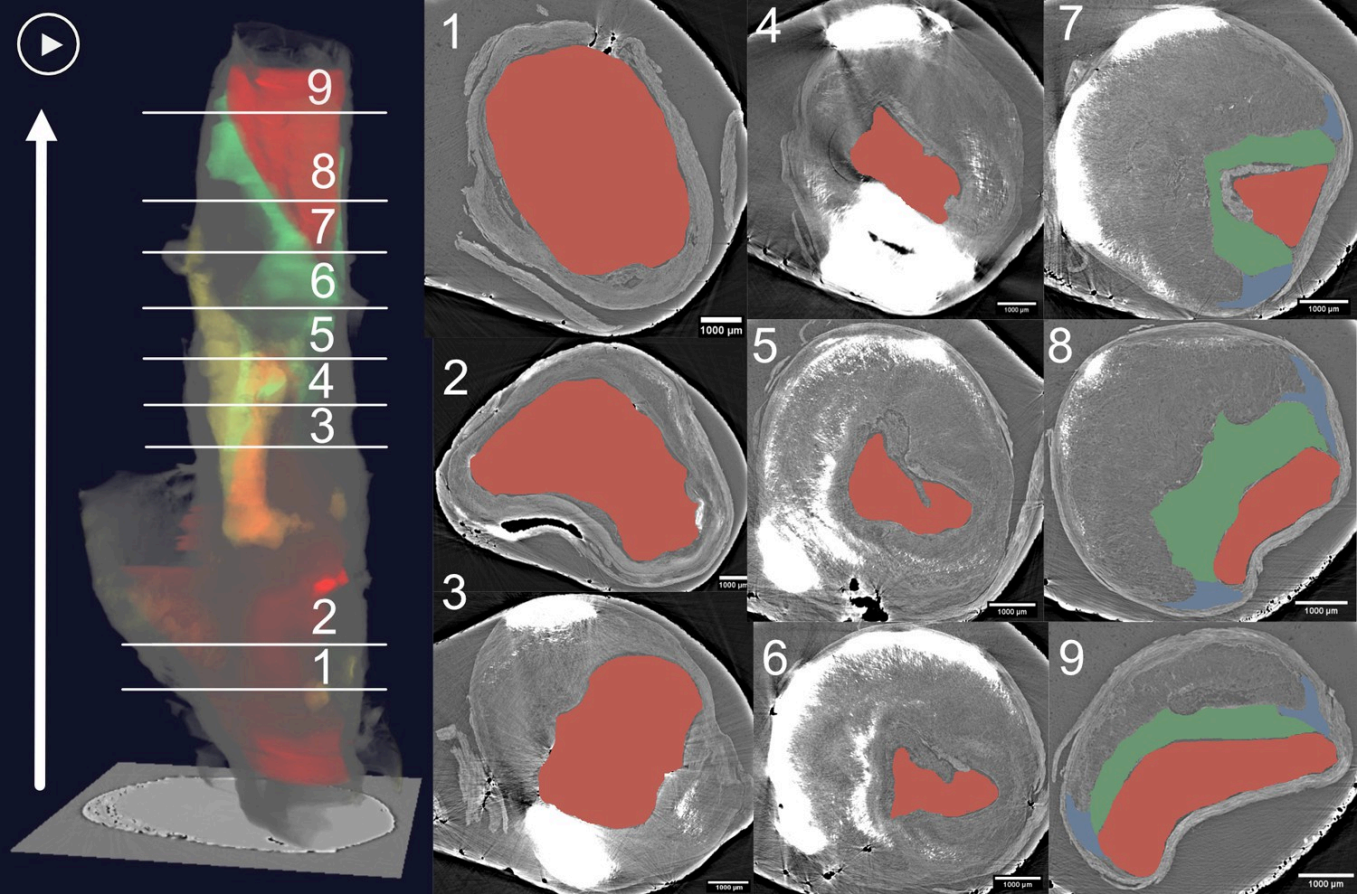
Irregular lumen (marked in red) and variation of fibrous cap thickness (marked in green) with 3D rendering. To the left is the 3D volume of a plaque with the calcifications, lumen, and fibrous cap segmented and highlighted with yellow, red, and green. The arrow indicates the direction of the blood stream. To the right, nine different axial planes with the lumen highlighted with red are shown to convey the lumen’s variation along the plaque length. The axial planes also show levels with different thickness of the fibrous cap (green) and the different shapes of the shoulder region (blue). In the axial planes, the calcifications are seen as white areas since the micro-calcifications were easier to see without the segmentation and yellow highlight. "Play" symbol: movie material showing the 3D rendering of the full plaque, with the lumen and fibrous cap segmented and highlighted (S10 Movie in S1 File). Plaque ID 2 in S1 Table in S1 File.

The LR SRμCT showed a considerable variation of luminal shape and stenosis within each plaque, very much like a clinical CT (but with much higher resolution), and the variation was assessed in both the tomographic axial image stacks and in 3D. In Fig 7 and S10 Movie in S1 File, the lumen is segmented and highlighted with red to show this variation. Additionally, considering that the fibrous cap integrity and thickness is one of the most important factors for rupture and occurrence of symptoms, we also segmented the fibrous cap in green. Interestingly we could demonstrate a huge variation of its thickness along the length of the plaque showing the complexity and heterogeneity of the disease even in a single sample of a single patient. Such techniques, that focus only in some areas of imaging or stainings are potentially losing a large amount of information potentially relevant for the patient.

### Internal elastic membrane

**Fig 8:**
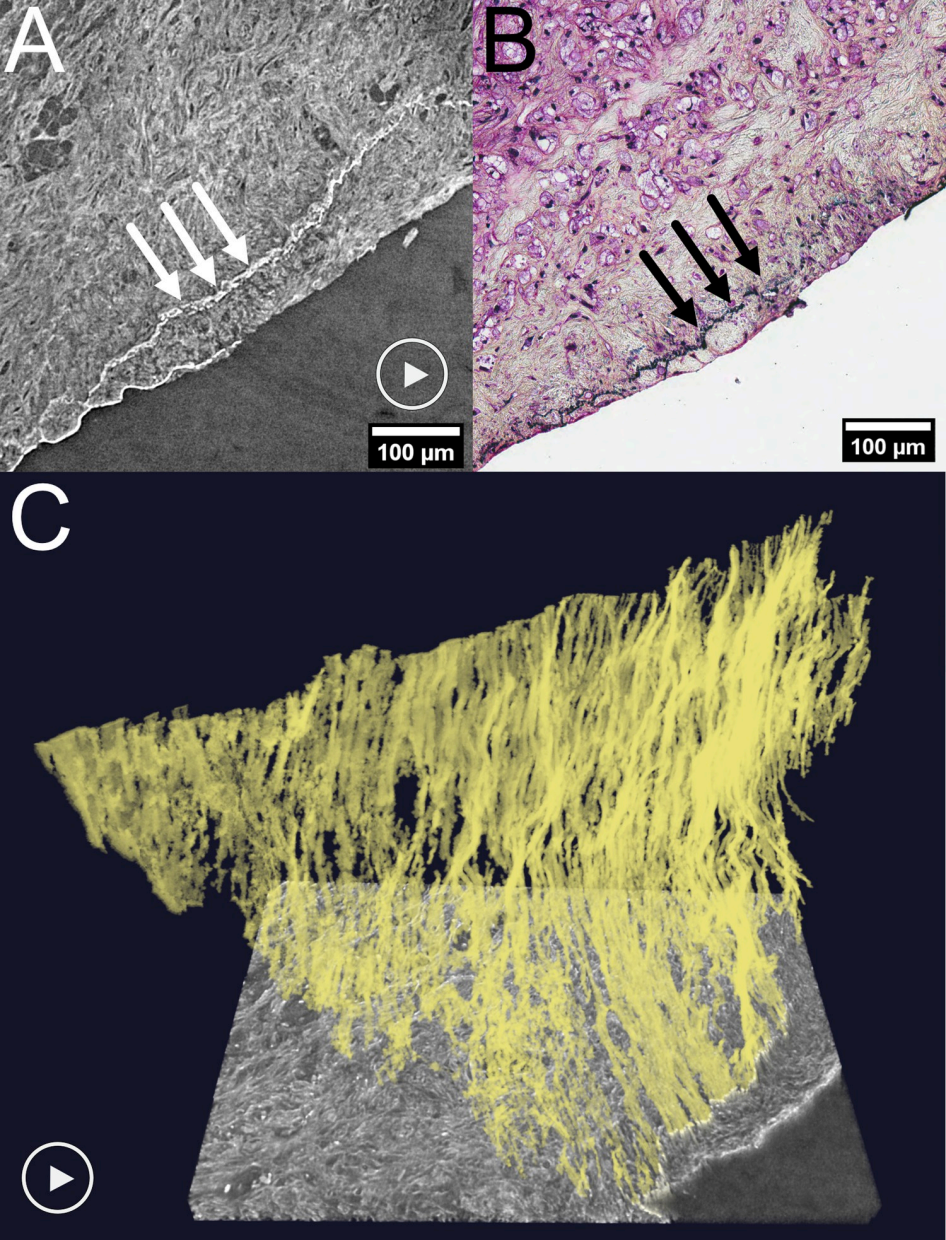
Internal elastic membrane depicted in 2D image stacks, histology and 3D rendering. HR SR μCT **(A)** and a histologic section with Movat staining (elastin in black) **(B)**. The arrows indicate the internal elastic membrane (white arrows in A and black arrows in B). **(C)** 3D reconstruction. "Play" symbols in **(A)** and **(C)** indicate movie material showing the membrane’s multiplanar course in the tomographic display (S11 Movie in S1 File) and the 3D rendering (S12 Movie in S1 File). Plaque ID 5 in S1 Table in S1 File.

Fig 8 displays the internal elastic membrane, a structure between the tunica intima and tunica media in the arterial wall. The membrane is easy to discern on HR SRμCT. It can be followed in the tomographic image sequence (S11 Movie in S1 File) and in 3D (S12 Movie in S1 File), where the complex three-dimensional structure of the membrane is visualized. In this example, the membrane is highly fragmented, which is an important pathologic process to allow the migration of smooth muscle cells from the media to the intima [18, 19].

## Discussion

This concept study demonstrates that synchrotron radiation μCT renders excellent resolution in the micron range in images of human carotid atherosclerotic plaques. This non-destructive method provides tomographic 3D datasets with low resolution (6.5 μm) and high resolution (0.65 μm) voxel sizes and excellent tissue contrast with the Paganin phase retrieval method in human plaques. In this initial study on five plaques from patients, we have obtained images of multiple typical plaque features, including ruptures, thrombus, neo-vascularization, inflammatory infiltrates, shoulder regions, LRNC, disrupted endothelium at the plaque-lumen interface, thin fibrous caps, calcifications, lumen irregularities, and internal elastic membrane fragmentation. The HR SRμCT was of such high resolution that comparison with histology (optimal optical resolution for histology is usually 0.2 μm in light microscopes) and analysis of microstructures was possible.

The non-destructive nature of whole plaque synchrotron radiation-based μCT, allows carotid plaque visualization with preserved integrity of the three-dimensionality of complex structures that are difficult to appreciate by 2D histology images. Complex pathological features such as ruptures, thrombi, LRNC, luminal irregularities, and the internal elastic membrane can be viewed in any plane and are readily visualized with 3D rendering. The 3D visualization of the plaque structure clearly illustrated the eccentric morphology of the plaque and the partly helical orientation of the components. This in in line with previous observations by Akyildiz AC et al [20] and in agreement with the dynamic interaction between the vessel wall fibers and the hemodynamic forces that they must withstand, even more in the advanced stages of the disease.

Histological and immunohistochemical methods are regarded as the gold standard to analyze plaque tissue *ex vivo* and provide much information about plaque morphology, but the mechanically and chemically destructive preparation process necessary to obtain histological images may compromise the integrity and composition and potentially confound the interpretation of the tissue specimen. A non-destructive method such as SRμCT that allows multiple re-scans can complement histology to mitigate such processing-induced artifacts and help understand plaque histopathology. In a future study it could be of interest to perform high-resolution SRμCT on plaque tissue at different stages of the histology preparation stages identify which stage is better suited for this type of imaging, as has been done on brain tissue by Rodgers et al. [21].

Scan time and image noise are significantly reduced in μCT with a synchrotron light source compared to a laboratory-based x-ray μCT. This allows for fast submicron imaging (within minutes), compared to the histological process that typically takes days to weeks. Synchrotron radiation-based and laboratory-based μCT of human atherosclerotic plaques have been conducted by other groups [10, 14, 22]. Still, to the best of our knowledge, this is the first time human carotid plaques have been imaged with phase-contrast μCT with 0.65 μm voxel resolution. In our opinion, the high-resolution volumes complement and facilitate comparison to histology in the study of atherosclerosis. Although access to synchrotron radiation can be limited, the utilization of the method is still important, since it shows images of plaques that have been handled minimally after surgical resection and therefore conveys a representative image of the shape and constitution of the atherosclerotic carotid plaque. The possibility of analysis in MPR (multiplanar reconstruction) and 3D visualization advances the perception and understanding of typical plaque structures, as those we have shown here.

Calcifications are generally a problem in histology, especially when sectioning. Commonly, tissues lacerate, and large calcifications become fragmented as well as get dislodged. In synchrotron phase-contrast μCT, macro-calcifications cause more absorption than surrounding non-calcified tissue leading to artifacts and an oversaturated image of the calcification. This can be reduced by adapting the dynamic range to very dense tissue, which would allow the visualization of details within the calcification. This method could complement histology, where large calcifications usually are challenging to preserve intact. (Fig 6). Histology is nevertheless even more time consuming due to numerous procedural steps demanding skilled human resources such as embedding, sectioning, fixation, dehydrations, infiltration, rehydration, mounting and staining.

Artifacts due to high sample density are less of a problem in sites with small calcifications, which allow us to image the distribution and morphological layout of microcalcifications. We generally found microcalcifications in thick fibrous caps and the interface between tunica media and necrotic areas in our material. The distribution of microcalcifications was overall consistent between SRμCT and histology, but even small calcifications could be seen dislodged in histology from their original position in the tissue. In situations where the calcifications had moved outside of the main plaque section, the disruption of original morphology was apparent. But, in histologic sections where the microcalcifications had moved within plaque margins, the change was less noticeable and better seen when compared to the congruent axial plane on HR SRμCT.

For our study, we used the established Paganin phase-retrieval method [23], assuming a constant delta-to-beta ratio throughout the sample. This method rendered excellent contrast in all plaque areas except in proximity to large calcifications or air bubbles entrapped in the paraffin embedding medium, as seen in the LR SRμCT in Fig 2A, where the assumption no longer holds.

μCT with 2048x2048x2048 voxels of 16-bit dynamic range generates large amounts of data when stitched into larger volumes. Hence, post-processing the volumes, especially when many volumes are stitched, requires high computational capacity. Depending on the number of stitched scans, the image stacks’ data size spanned from 12.5 Gb to 260.6 Gb (S1 Table in S1 File).

SRμCT scans with these voxel sizes and coverages take a relatively long time to obtain. Each single ROIs scan takes 2 min 04 s for LR SRμCT and 6 min 33s for HR SRμCT. Scanning the full plaque volume in LR can take approx. 22 minutes (depending on the length of plaque), while a region of interest in HR SRμCT could range between 6 minutes to 96 minutes (depending on area size). Although scan times with SRμCT is much shorter than the histologic processing, access to synchrotron facilities may be slow and highly competitive. Hence, the gain is not processing time but the possibility of non-destructive and volumetric imaging with high resolution.

Imaging of human carotid plaques with SRμCT can bridge the resolution gap between clinical CT (sub-millimeter pixel size) and histology (μm pixel size) and advance the understanding and interpretation of clinical CT. In general, applying a non-destructive method such as SRμCT makes it easier to translate histopathological findings into CT images. The technique can potentially also help us interpret CT findings in a clinical setting, in multiple organ systems and pathologies. Further, medium resolution microtomography systems can be used for pre-scanning a larger number of samples, aiding the selection of samples for detailed analysis by synchrotron experiments or histology, as suggested by e.g. Nguyen et al. [24]. And as image quality of microtomography systems based on laboratory X-ray tubes is increasing, it is conceivable that some studies can be performed without the use of synchrotron radiation or histology at all.

SRμCT has several limitations, such as the inability to stain the tissue for specific components, which is inherent to its non-destructive nature. On the other hand, it allows a very high resolution and visualization of untouched structures (avoiding procedural artefacts), that can then be recognized after performing this type of study comparing with histology/immunohistochemistry.

Due to limitations in allocated beam time combined with our intention to scan as many various plaque pathologies as possible, the HR SRμCT did not cover each plaque full length and surface. The limitations of available beamtime with synchrotron radiation would make a laboratory-based system, capable of reaching similar resolution, an asset. Such a system is easier to access, which would be ideal for research purposes. Still, laboratory-based systems could also potentially introduce other limitations: The longer the scan, the higher the risk for minimal movements that could affect the final result. Also, histologic sections with large calcifications or LRNC were often lacerated, and the plaque shape often slightly changed during sectioning, which limited the number of histologic sections that could be matched to SRμCT.

The largest advancements of our study (comparing to the previous literature [10, 14, 22]) are: 1) the use of more carotid samples from different living patients (not fragments from the same sample), 2) assessed with 3 immunostainings and 2 histological stainings (one of them a pentachrome providing 5 components), 3) depicted with the highest resolution so far due to the combination of a propagation-based phase-contrast method with the synchrotron source. In addition, we performed both low- and high-resolution scans on the samples, using simple embedding, allowing us both a global screening of the sample, as well as zooming in to regions of particular clinical interest without destroying the tissue and in 3D.

In conclusion, SRμCT with a submicron voxel size is an excellent non-destructive method to image clinically relevant features of the atherosclerotic plaques. The method renders the viewer high resolution, multiplanar and 3D images of microstructure morphology and could advance the knowledge of the microstructural architecture of human atherosclerosis. In our study, the SRμCT was compared to histology to validate the different morphological structures. Still, even without histology, many of the plaque-typical pathological structures such as plaque rupture, thrombus, and lipid rich necrotic cores, could be identified and analyzed in detail, showing the value of SRμCT for plaque characterization.

## Supporting information

S1 File (PDF)
