# Supplementary material for: Sub-micrometer morphology of human atherosclerotic plaque revealed by synchrotron radiation-based μCT—A comparison with histology
Source: PLoS One. 2022 Apr 26;17(4):e0265598. doi: 10.1371/journal.pone.0265598 (PMC9041845; doi:10.1371/journal.pone.0265598)
Supplement: S1 File — (PDF) [file pone.0265598.s001.pdf]

Supplementary Information for:

Sub-micrometer morphology of human atherosclerotic plaque revealed by synchrotron radiation-based μCT - a comparison with histology

My Truong[1¶], Till Dreier[2,3¶], Johan Wassélius[1], Lena Sundius[4], Ana Persson[4], Goran Lovric[5,6], Anne Bonnin[6], Isabel Goncalves[7&], and Martin Bech[2&*]

[1] Diagnostic Radiology, Department of Clinical Sciences Lund, Lund University, Skåne University Hospital, Lund, Sweden

[2] Department for Medical Radiation Physics, Clinical Sciences Lund, Lund University, Lund, Sweden

[3] Excillum AB, Kista, Sweden

[4] Clinical Sciences Malmö, Lund University, Malmö, Sweden.

[5] Center for Biomedical Imaging, École Polytechnique Fédérale de Lausanne, Lausanne, Switzerland.

[6] Swiss Light Source, Paul Scherrer Institute, Villigen, Switzerland

[7] Cardiology, Skåne University Hospital and Department of Clinical Sciences Malmö, Lund University, Lund, Sweden

* Corresponding author

E-mail: martin.bech@med.lu.se (MB)

¶ These authors contributed equally to this work.

& These authors also contributed equally to this work.

This PDF file includes:

Supplementary text

Figures S1 and S2 with legends

Tables S1 and S2

List of supplementary movies available online.

Detailed immunohistochemistry and histology.

For macrophage staining, the primary monoclonal antibody mouse anti-human CD68, clone KP1 (DakoCytomation, Glostrup, Denmark) was diluted to 1:800 in 1% bovine serum albumin (BSA) in Tris-buffered saline (TBS) wash, ImmPACT DAB Peroxidase Substrate (Vector Labs, CA, USA) and MACH 3 Mouse HRP-polymer detection (Histolab, Askim, Sweden). Counterstaining was with Mayer's Hematoxylin.

For detection of vascular smooth muscle cells, a primary antibody monoclonal mouse anti-human smooth muscle cells actin clone 1A4 (Dako Cytomation, Glostrup, Denmark) was used, diluted to 1:800 in 1% BSA stain buffer and TBS wash and ImmPACT DAB Peroxidase Substrate (Vector Labs, CA, USA) and MACH 3 Mouse HRP-polymer detection (Histolab, Askim, Sweden). Counterstaining was Mayer's Hematoxylin.

We used the primary antibody monoclonal mouse anti-human glycophorin A (CD235a) clone JC159 (DakoCytomation, Glostrup, Denmark) to visualize erythrocytes. The antibodies were diluted 1:400 in BSA, ImmPACT DAB Peroxidase Substrate (Vector Labs, CA, USA), and MACH 3 Mouse HRP-polymer detection (Histolab, Askim, Sweden). Counterstaining was done with Mayer's Hematoxylin.

For the assessment of calcifications, the von Kossa stain was used. Sections were fixated in absolute alcohol and then rinsed. 1% silver nitrate solution in a clear glass Coplin jar was placed under ultraviolet light for 20 minutes. A 5% sodium thiosulfate solution was used for 5 minutes to remove the unreacted silver after rinsing. After rinsing, a counterstain with 0.1% nuclear fast red solution was used for 5 minutes. After rinsing, dehydration, and clearing in xylene, mounting was made with the mounting medium Pertex ® (Histolab, Askim, Sweden).

For the assessment of collagen, elastin, fibrin, mucin, nuclei, and muscle, Movat Pentachrome stain was used. The section was deparaffinized with xylene, 98%, and 70% alcohol and then rinsed. Alcian Blue was added for 15 minutes. After washing and rinsing, Hematoxylin solution was added for 15 minutes. After rinsing, the section was differentiated in 2% aqueous ferric chloride and then rinsed. Sodium thiosulfate 5% was added for one minute, and then the section was washed and rinsed before Crocein scarlet-acid fuchsin was added for 3 minutes. After rinsing with water, the section was then rinsed in 0.5% acetic acid water for 30 seconds before 5% aqueous phosphotungstic acid was added for two sessions, each lasting 10 minutes. Afterward, the section was rinsed in 0.5% acetic acid water for 30 seconds and Absolute alcohol three times (three changes) for one minute each time. Lastly, the section was stained with 6% Alcoholic Saffron for 15 minutes and rinsed in Absolute Alcohol three times (three changes) and twice in xylene. For mounting, Pertex mounting medium was used.
All stained sections were scanned in 20x magnification with Aperio ImageScope 12.3.2.8013 (Leica Biosystems Inc. Buffalo Grove, IL, USA)

Figure S1: Sample mounting and alignment

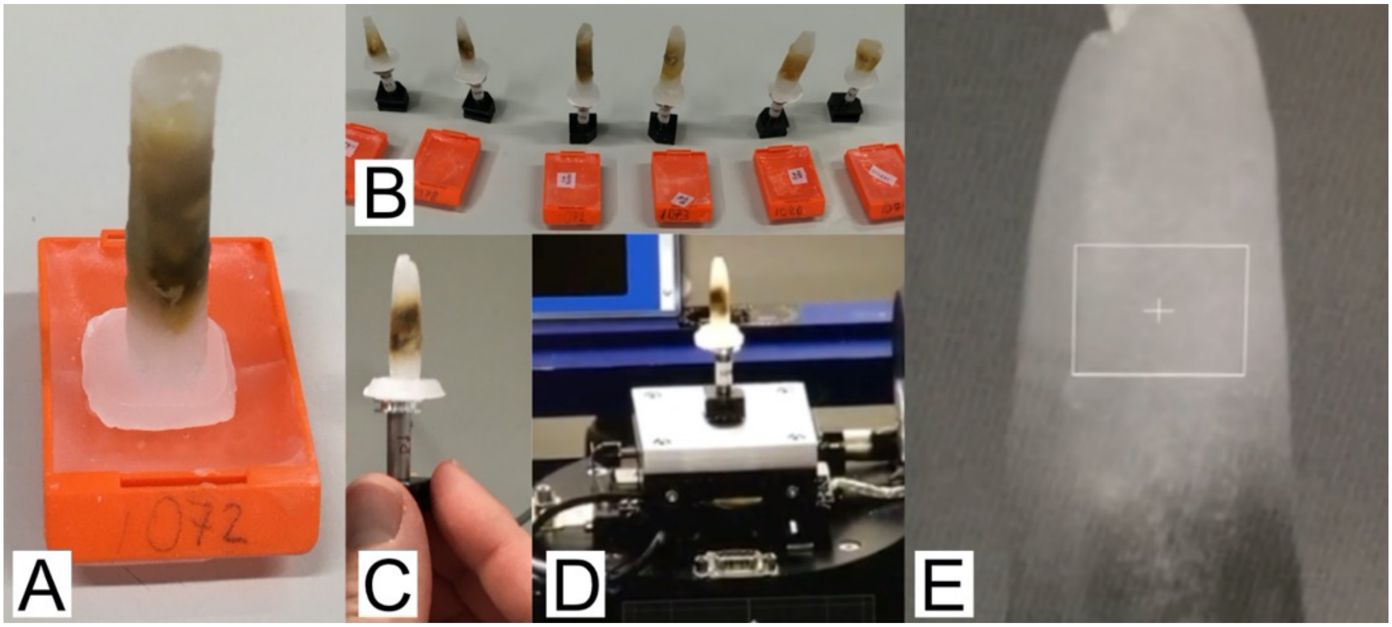

Fig S1. Embedded samples, mounting and alignment. An example of a paraffin-embedded plaque is shown in **(A)** In **(B)**, all five plaques mounted on pins are displayed (the 6th plaque to the right was never scanned). In **(C)**, a mounted plaque is seen up-close, and in **(D)**, the plaque is placed in the tomography setup at the beamline. **(E)** shows an image of the plaque taken from an optical camera used for pre-aligning the sample before the tomographic scan.

Figure S2. Calcifications

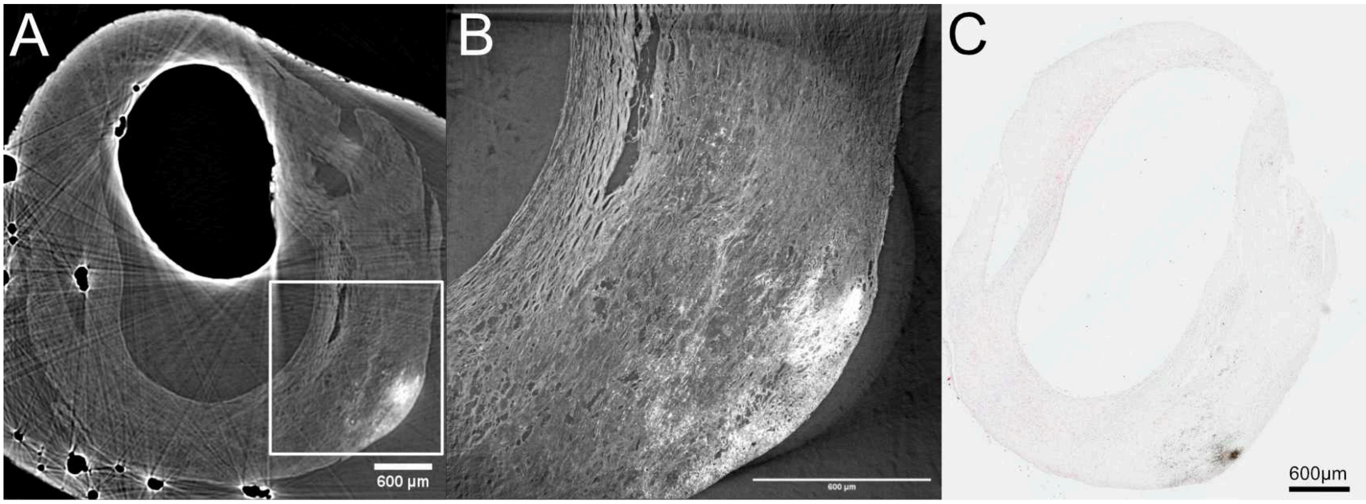

Fig. S2. Calcifications. (A) LR SR μCT of an axial section plane with microcalcifications seen as irregular white dots with coalescence with a gradual change to macrocalcification (>100μm) within the marked area. The box in **(A)** marks the area scanned with HR SRμCT seen in **(B)**. In **(C)**, the corresponding histologic section stained for calcifications (von Kossa) is seen (black dots).). Plaque ID 3 in Table S1.

Table S1. Imaging data

Plaque ID	Dimensions stitched LR SRµCT (pixels)	Number of stiched LR SRµCT	Dimensions stitched LR SRµCT (mm)	HR Level	Number FoV of scans	Dimensions HR SRµCT (pixels)	Dimensions stitched HR SRµCT (mm)	Size (Gb)
**1**	4860x2048x2048	11	31.59x13.26x13.26					37.7
				1_1	4 (2x2)	2161x4391x4384	1.41x2.85x2.85	77.5
				1_2	4 (2x2)	2160x4388x4383	1.41x2.85x2.85	77.4
				1_3	4 (2x2)	2161x4385x4406	1.41x2.85x2.86	77.8
**2**	4860x2048x2048	11	31.59x13.26x13.26					37.7
				2_1	16 (4x4)	2162x8052x8037	1.41x5.23x5.23	260.6
				2_2	4 (2x2)	2161x4383x4409	1.41x2.85x2.87	77.8
**3**	1619x2048x2048	4	10.52x13.26x13.26					12.5
				3_1	1	2060x1560x2560	1.34x1.01x1.66	26.4
**4**	3527x2048x2048	8	22.93x13.26x13.26					27.3
				4_1	4 (2x2)	2160x4389x4413	1.40x2.85x2.86	77.9
**5**	4860x2048x2048	11	31.59x13.26x13.26					37.7
				5_1	4 (2x2)	2162x4398x4391	1.40x2.86x2.85	77.8
				5_2	4 (2x2)	2161x4384x4396	1.40x2.85x2.86	77.6

Table S2 SR µCT parameters

	Low-resolution scan	High-resolution scan
**Camera**	pco.edge 4.2 sCMOS	pco.edge 5.5 sCMOS
**Resolution**	2048x2048	2560x2160
**Pixel size (µm)**	6.5	6.5
**X-ray camera optics**	1x	10x optics
**Effective pixel size (µm)**	6.5	0.65
**Scintillator**	300 µm LuAg:Ce	20 µm LuAg:Ce
**Beam Energy (keV)**	21	21
**Beam geometry**	Parallel	Parallel
**FoV at sample position**	570 pixels * 6.5 µm = 3.705 mm vertically<br><br>2048 pixels * 6.5 µm = 13.260 mm horizontally	2560 pixels * 0.65 µm = 1.664 mm vertically<br><br>2160 pixels * 0.65 µm = 1.404 mm horizontally
**Sample rotation (degrees)**	180	180
**Absorbers, Filters**	5 mm pyrolitic graphite, 400 µm Al, 10 µm Fe	100 µm Al, 10 µm Fe
**Projections**	1501	1501
**Exposure time (ms)**	30	200
**Sample-Detector distance (mm)**	3300	55
**Flat-field images**	100	100
**Dark-field images**	30	30
**Scan time per CT (min:s)**	2:04	6:33

List of supplementary movies available online.

S1 Movie. Full plaque volume with 3D render

https://osf.io/73xpd/

S2 Movie. LR SR μCT of full plaque volume

https://osf.io/vhm7g/

S3 Movie. HR SR μCT of full plaque transversal area

https://osf.io/j85cq/

S4 Movie. LR SR μCT full plaque volume with rupture

https://osf.io/7a3ht/

S5 Movie. LRNC content exposed to lumen with thrombus formation

https://osf.io/8ur4c/

S6 Movie. HR SR μCT image stack of organized thrombus

https://osf.io/esryf/

S7 Movie. 3D render of organized thrombus with neo-vessels

https://osf.io/x8fyc/

S8 Movie. Shoulder region shown with HR SRμCT

https://osf.io/6uf3d/

S9 Movie. LRNC with HR SRμCT

https://osf.io/mkuj4/

S10 Movie. 3D rendering with the lumen, fibrous cap, and calcifications segmented and highlighted with red, green, and yellow

https://osf.io/q3t2y/

S11 Movie. Image stack of the internal elastic membrane.

https://osf.io/2fvbp/

S12 Movie. The internal elastic membrane in 3D rendering.

https://osf.io/du3fs/
